# RETRACTION: Ointment of *Brassica oleracea* var. *capitata* Matures the Extracellular Matrix in Skin Wounds of Wistar Rats

**DOI:** 10.1155/ecam/9834570

**Published:** 2025-11-14

**Authors:** Evidence-Based Complementary and Alternative Medicine

RETRACTION: M. M. Sarandy, R. D. Novaes, S. L. P. da Matta, et al., “Ointment of *Brassica oleracea* var. *capitata* Matures the Extracellular Matrix in Skin Wounds of Wistar Rats,” *Evidence-Based Complementary and Alternative Medicine* (2015): 919342, https://doi.org/10.1155/2015/919342.

The above article, published online on 11 June 2015 in Wiley Online Library (https://wileyonlinelibrary.com), has been retracted by John Wiley & Sons Ltd. The retraction has been agreed due to figure concerns, raised by Fabian Wittmers, as well as on PubPeer [1]. On further investigation, the below concerns have been identified:

• In the Day 4 column of Figure 1, the Sunflower and Ointment panels appear to contain repetitive elements.
• In Figure 1, the Control panel for Day 4 appears identical to the Sal panel for Day 7 (F1) in Figure 3 of [2].
• In Figure 1, the Sunflower panel for Day 4 appears identical to the LE10 panel for Day 7 (F1) in Figure 3 of [2].
• In Figure 1, the Ointment panel for Day 4 appears identical to the LE5 panel for Day 7 (F1) in Figure 3 of [2].
• In Figure 1, the Day 0 panels appear identical to the Day 0 panels of [3], despite representing different conditions.
• In Figure 1, the Day 8 panels appear identical to the Day 8 panels of [3], despite representing different conditions.
• In Figure 1, the Day 16 panels appear identical to the Day 16 panels of [3], despite representing diffferent conditions.
• In Figure 1, the Day 20 panels appear identical to the Day 20 panels of [3], despite representing different conditions.
• Figure 2(a) appears similar, when rotated and resized, to the L30/Day 7 and L90/Day 14 panels in Figure 2 of [4].
• In the Day 4 column of Figure 3, the control and Balsam panels appear to contain repetitive elements.
• In the Day 20 column of Figure 3, the Sunflower and Balsam panels are identical.

After assessment of the author's response and the concerns, the results are no longer reliable and the article is retracted.

The authors did not respond to the retraction notice.
